# Designing a pharmacist primary care certificate training program based on employer perceptions

**DOI:** 10.1016/j.rcsop.2022.100191

**Published:** 2022-10-10

**Authors:** Kelsey D. Frederick, Rachel E. Barenie, M. Braden Dill, James S. Wheeler

**Affiliations:** a University of Tennessee Health Science Center College of Pharmacy, 301 S. Perimeter Park Drive, Suite 220, Nashville, TN 37211, United States; b University of Tennessee Health Science Center College of Pharmacy, 881 Madison Avenue, Memphis, TN 38163, United States; c University of Tennessee Health Science Center College of Pharmacy, 1924 Alcoa Highway, Box 117, Knoxville, TN 37920, United States

**Keywords:** Pharmacist, Primary care, Certificate, Continuing professional development, Continuing education, Employer perceptions

## Abstract

**Background:**

As the pharmacy profession transforms toward practice centered around direct patient care and clinical services, upskilling the existing workforce may be required for pharmacists to take on expanded roles, especially in an increasingly competitive job market.

**Objective:**

To explore pharmacist employer perceptions of a primary care certificate training program including its design, value, and relevance and to develop and implement a pharmacist primary care certificate training program based on study results.

**Methods:**

Focus groups were conducted to a point of saturation in December 2020 via video conference. Participants were identified via the study institution's continuing professional development registrant listserv and invited to participate via self-selection. Interviews were recorded, transcribed, and underwent inductive thematic analysis.

**Results:**

Four focus groups were conducted with 15 pharmacist employers. Employers perceived primary care certificate training as valuable, helping pharmacists sustain shifting roles and increasing opportunities in a competitive job market. A combination of clinical and practice management topics with emphasis on an experiential component was recommended to achieve expected competency levels and favorably influence hiring decisions. The primary care certificate was specifically recommended to pharmacists aiming to transition into primary care or for pharmacists who did not complete residency training.

**Conclusions:**

This study's findings informed development of a pharmacist primary care certificate program containing didactic and experiential training on a variety of key topics. As pharmacists' roles evolve, this program may prepare pharmacists to engage in direct patient care and develop skills and expertise necessary to succeed in outpatient primary care.

## Highlights

• Employers prioritize and expect training beyond terminal degrees to bridge the gap between education and practice.
• Employers valued primary care certificate programs for making candidates more marketable and influencing hiring decisions.
• A primary care certificate program will prepare pharmacists to develop skills to succeed in outpatient primary care.

## 1 Introduction

Primary care is at a crossroads in the United States triggered by growth in specialization, low payments for preventative healthcare services, and primary care physician demand-capacity mismatch.^1^, ^2^, 3., 4., 5, 6 Pharmacists' roles are expanding, as evidenced by their engagement in direct patient care roles via collaborative pharmacy practice agreements, providing comprehensive medication management services, and offering preventive care services, such as vaccines or point-of-care testing. Many community pharmacies have transformed into a wellness hub model, expanding beyond traditional medication dispensing services by incorporating primary care clinics into their facilities and offering medication management services.^7^^,^^8^ Pharmacists improve the health and wellness of patients within the primary care setting by ensuring the safe and effective use of medications, performing patient assessments, developing collaborative practice agreements, and monitoring medication-related lab results.^9^ Further, the principles of pharmacy education, while still including mastery of skill in preparation and dispensing of medications, now expand to all-encompassing pharmaceutical care responsibilities.^10^ Thus, it is crucial for pharmacists to maintain competence through engaging in self-assessment, lifelong learning, continuing pharmacy education, and continuing professional development.^11^

One significant way in which pharmacists can demonstrate sustained competence and engage in professional development is by completing certificate programs that offer formal seminars to develop the necessary skills to become specialized within various areas of pharmacy practice.^12^^,^^13^ Certificate programs that meet continuing pharmacy education (CPE) accreditation standards are defined as activities that “systematically acquire specific knowledge, skills, attitudes, and performance behaviors that expand or enhance practice competencies.”^14^ Formats of certificate programs must include a didactic component, a practice component, and should be a minimum of 15 contact hours.^11^ Previous literature has demonstrated completion of certificate programs can help pharmacists excel within their current positions or become more competitive in the eyes of potential employers when applying for future job positions.^15^, ^16^, ^17^ Other more traditional routes to increase pharmacist marketability exist, including completing a residency, fellowship, or an advanced degree; however, these options may not be feasible for everyone due to a variety of factors (e.g., cost, duration, and limited residency programs available for match placement). A more affordable option is to complete a certificate program, taking weeks to months to complete instead of years.

By pursuing primary care certificate training, pharmacists may prepare for their advancing roles in providing patient care. It is projected that there will be a surplus of pharmacists as the number of graduates exceeds the expected annual pharmacist job openings throughout the upcoming decade.^18^ Furthermore, there is a proposed need to redefine the primary care physician workforce shortage to a demand-capacity mismatch.^6^ In their 2013 Health Affairs commentary, Bodenheimer and Smith argue that primary care practices could increase capacity to meet clinical demand if they reallocate many clinical responsibilities to non-physician team members—like pharmacists.^6^ A primary care certificate may provide pharmacists across various pharmacy settings (e.g., hospital, ambulatory care, community pharmacy, etc.), degrees and training (e.g., bachelor's degree, Doctor of Pharmacy, residency, specialization, other certification, etc.), and experiences with the essential skills needed to meet expanding pharmacy roles and distinguish themselves within the competitive pharmacist job market. Thus, to ensure primary care certificate training meets the needs of the profession, pharmacists, and employing organizations, pharmacist employer insights are greatly valued in informing the design of such a certificate program. There is not robust evidence describing the need for or optimal design of a primary care certificate for pharmacists; the current gap is unclear. Therefore, we set out to collect pharmacist employer perceptions to generate this evidence through our research.

The primary objective of this study was to explore the perceptions that pharmacist employers and hiring managers have toward a pharmacist primary care certificate training program, including their opinions regarding the design of the program, assessment methods, support for the program, perceived value of the program, and the general need for the development of this program. The secondary objective of this study was to utilize the results of employer focus groups to design and implement a pharmacist primary care certificate training program at the study institution based on employer perceptions and recommendations.

## 2 Methods

Focus groups were conducted virtually in December 2020 to explore pharmacist employer perceptions of primary care certificate training and their recommendations for optimal program design.^19^ Focus group methodology was selected instead of individual interviews to facilitate group-thinking and interaction between participants and to explore and obtain rich data and collective group perceptions.^19^ Employers also had the opportunity to participate in a survey collecting their perceptions of certification training and optimal primary care certificate program design. These quantitative survey results are presented in a separate manuscript. Qualitative focus group data was collected to give employers an opportunity to elaborate on their perceptions and recommendations of primary care certificate training for pharmacists. Participants were identified via purposive sampling according to the study institution's continuing professional development (CPD) registrant listserv and invited to participate via self-selection. Focus group participants were all members of the study institution's Office of CPD listserv, which is used to announce upcoming CPD programming and other office news. Individuals were identified and invited to participate in the study via self-selection if they met the following criteria: contributed to hiring or employment decisions within their current role and organization. If interested in participating, individuals were asked to submit availability for focus group participation via an online survey interest form. The focus group interview guide (Appendix A) was semi-structured and developed by the research team members and included questions about perceived value of certificate training in today's job market, most important primary care competencies expected for pharmacists to achieve via certificate training, recommendations for content delivery and assessment, and other essential design elements for a primary care certificate training program to favorably influence the employer's decision to hire a pharmacist. No preconceived constructs or themes were determined prior to data collection. Prior to this study, there were no certificate programs offered by the study institution's Office of CPD. Three of the four researchers (KDF, REB, JSW) are faculty within the study institution's Office of CPD who administer continuing education programming for pharmacists and other healthcare professionals. At the time of data collection, analysis, and manuscript writing, the remaining author (MBD) was a fourth-year student at the study institution.

Informed consent was obtained verbally from each participant prior to the focus group. Focus groups were conducted and audio-recorded by the primary author until a point of thematic saturation, at which no new information was collected.^19^ Field notes were made during and after focus groups and considered during analysis.^20^ No individuals other than the participants and researchers were present at the time of the focus groups. Recordings were transcribed verbatim by a third-party service and underwent inductive thematic analysis by a two-coder team of researchers (KDF and MBD) trained in qualitative methods to capture and represent themes from the data after data collection occurred (NVivo, Melbourne, Victoria, Australia).^21^ The researchers first coded transcripts separately then met to discuss the codes and themes until consensus was reached in order to avoid introducing researcher bias and to accurately denote themes as one coding system.^21^ The consolidated criteria for reporting qualitative research (COREQ) checklist was referenced throughout data collection and analysis.^22^ Following data collection and analysis, results were utilized to design, develop, and implement a pharmacist primary care certificate training program at the study institution based on employer perceptions and recommendations. Approval of this research was granted by the study institution's Institutional Review Board (20–07814-XM).

## 3 Results

Four focus groups were conducted with 15 pharmacist employers who reported contributing to hiring decisions within their respective organizations. Focus groups ranged from 62 to 72 min and 2 to 5 participants per group. Most participants reported employment in the community or ambulatory care setting and one to five years of experience in their hiring role (see Table 1 for participant demographics). There were no participants who dropped out of the study. No repeat focus groups were conducted.

**Table 1:** Participant demographics.

Demographics	Number (Percent) of Participants out of 15
Licensed pharmacist	
Yes	15 (100%)
No	0 (0%)
Pharmacy practice setting	
Hospital	1 (7%)
Ambulatory Care	6 (40%)
Community	7 (46.67%)
Other (Specialty infusion pharmacy)	1 (7%)
Location of practice site	
Tennessee	13 (86.67%)
Outside Tennessee	2 (13.33%)
Years working in pharmacist hiring role	
<5 years	3 (20%)
5 to 10 years	6 (40%)
>10 years	4 (26.67%)
Years since graduating pharmacy school	
5 to 10 years	5 (33.33%)
>10 to 20 years	4 (26.67%)
>20 years	5 (33.33%)
Terminal pharmacy degree	
BS Pharm	3 (20%)
PharmD	12 (80%)
Age	
<30 years	1 (7%)
30 to <40 years	3 (20%)
40 to <50 years	6 (40%)
50 or more years	5 (33.33%)
Sex	
Male	7 (46.67%)
Female	8 (53.33%)
Race	
White	13 (86.67%)
Chinese	1 (7%)
Asian Indian	1 (7%)

Three overarching themes were derived from the data and all three reveal that pharmacist employers perceive primary care certificate training as valuable, helping pharmacists sustain shifting roles and responsibilities within the profession and increasing the spectrum of opportunity for pharmacists in a competitive job market. See Table 2 for a summary of these themes.

**Table 2:** Summary of qualitative themes.

Themes	Main Points	Sample Quotes
Theme 1: Pharmacist Employers Support the Need for Pharmacist Primary Care Certificate Training	• This certificate may prepare pharmacists to engage in direct patient care and develop skills and expertise necessary to succeed in outpatient primary care and sustain expanding roles • This certificate may bridge the gap between pharmacy education and practice • Employers recommended the certificate for pharmacists, especially those aiming to transition into primary care or who did not complete residency training • Most employers were willing to sponsor their employees' pursuit of this certificate	“I feel like pharmacy role is being pushed to doing more collaborative practice and adherence training, things like that. It's not just filling prescriptions.” (Participant 2) “I think we're at a state in pharmacy of transition right now. And we're going to have to move into more of a clinical role. If we don't develop an outreach to primary care through certificate programs or some method, then I think not only is it going to hurt the communities in rural America, it's going to hurt the colleges, the educational institutions.” (Participant 6) “I think it helps bridge the perceived disconnect between what pharmacy school feels like and what the real world [of pharmacy] feels like.” (Participant 8) “I see it more envisioned for the person who tried for the residency, but didn't match, but still is wanting something more and wanting to show that they are increasing their skill set and their knowledge and not just biding time to try to rematch the next year.” (Participant 4) “I've been out [of school] for 25 years. I don't have the feeling I could go back and do a residency. I could go back and do something to make myself more relevant, dedicate 30 to 40 h of study to take that next step.” (Participant 7) “I think it's a great opportunity for someone who has chosen a focus and then wants to move on. So, let's say they had chosen hospital and want to move to retail, or chosen retail and want to move to hospital, it gives them an opportunity to say, ‘Hey, what things do I need to know in that practice area?’ and then be more prepared for going into that new job.” (Participant 15) “Coming from my background with independent [pharmacy], having a certificate would be one step toward trying to step out a little more from the regular retail pharmacists, like me, that didn't have residencies. So that's one thing that would be beneficial for an independent [pharmacist] like me who wants to have a niche or do more collaborating.” (Participant 2) “I simply would [sponsor pharmacists' pursuit of this certificate] because I think it's a return on investment. It does bring value to our stores, to our patients, and hopefully lift the profitability of our stores.” (Participant 10) “I would recommend it as well. I think the more likely opportunity I would be to be recommending it is that pharmacist who says, ‘I'm stuck in this job. What I'd really like to do is this. What can I do to make myself more marketable at that next job opportunity that I see?’” (Participant 15)
Theme 2: Pharmacist Employers Value Primary Care Certificate Training Experience When Making Hiring Decisions	• Employers perceived the certificate as valuable in helping pharmacists sustain shifting roles and increasing job opportunities • A pharmacist who completes this certificate may be a more competitive job candidate • A pharmacist who completes this certificate may bring more value to the overall organization	“I think it's a very, very, very valuable thing. For new graduates who are having a hard time finding jobs, this may be a good route to further education increase their spectrum.” (Participant 11) “What I tell students is that you've got to differentiate yourself from the masses that are graduating right now. If you can't do that personality-wise, you've got to have some kind of training that elevates you from the group.” (Participant 10) “Having a certificate would indicate an additional level of commitment and motivation. It shows initiative and wanting to grow and implement new ideas that could be overall beneficial to the company.” (Participant 1) “It would certainly put them a leg up over someone who is more generalized just coming out of pharmacy school.” (Participant 15)
Theme 3: Recommended Primary Care Certificate Design According to Pharmacist Employers	• A combination of knowledge- and experiential-based learning • A combination of clinical and practice management topics • Clinical topics recommended include diabetes, hypertension, hyperlipidemia, anticoagulation, and chronic lung disease • Practice management topics recommended include billing and reimbursement, collaborative practice agreements, and communication	“60% hands-on, 40% didactic. You can learn so much through the didactic but until you actually apply it, sometimes it really doesn't stick.” (Participant 4) “I think experiential assessments are paramount. I don't think it's exclusively about knowledge. There are plenty of people who can take an exam and not take care of a patient. So, you need to be able to prove at the end of whatever the thing is that you can do what the paper says you can do. So, there would be some OSCE or some evaluation that shows a skill as well as the knowledge.” (Participant 13) “I think it's really important that these kinds of specific courses, for someone who doesn't have previous [primary care practice] experience, have an experiential component. I'm really excited to see how we can bridge that gap.” (Participant 3)

### 3.1 Theme 1: pharmacist employers support the need for pharmacist primary care certificate training

Participants provided reasons they believed a primary care certificate for pharmacists is needed and how it addressed an existing need in the pharmacy workforce or bridged an existing gap. This included employer perceptions related to evolving pharmacist responsibilities and growing opportunities and expectations for pharmacist collaboration and direct patient care. Specifically, participants noted that the role of pharmacists is being pushed to evolve and include more medication therapy and disease state management, collaborative practice, and not just filling prescriptions. Furthermore, pharmacist employers stated that a primary care certificate could help bridge the perceived gap between pharmacy education and practice.

*“I think we're at a state in pharmacy of transition right now. And we're going to have to move into more of a clinical role. If we don't develop an outreach to primary care through certificate programs or some method, then I think not only is it going to hurt the communities in rural America, it's going to hurt the colleges, the educational institutions.” (Participant 6).*

Furthermore, employers shared their perceptions of the ideal or apparent group of pharmacists who could benefit from this certificate training program. For example, the primary care certificate training may be valuable for pharmacists who did not complete residency training and/or were wanting to expand their primary care knowledge and skills for their current or future job position. Specifically, participants believed primary care certificate training could be valuable to and pursued by pharmacists who attempted but did not match with a residency program and still wanted to invest in training and strengthen their primary care skillset in preparation for their next career opportunity or to go through the residency match process again the following year. According to focus group participants, a primary care certificate may also be desirable to and pursued by pharmacists who may have graduated several years ago and who do not feel they could go back to complete a year-long residency but were seeking additional training to improve their skills. The training could also be beneficial for someone transitioning between jobs or roles.

*“I think it's a great opportunity for someone who has chosen a focus and then wants to move on. So, let's say they had chosen hospital and want to move to retail, or chosen retail and want to move to hospital, it gives them an opportunity to say, ‘Hey, what things do I need to know in that practice area?’ and then be more prepared for going into that new job.” (Participant 15).*

Participants shared that their perceived benefits of this certificate experience would not only be afforded to pharmacists who complete the program but also to their employing organizations, patients, and the profession.

*“Coming from my background with independent [pharmacy], having a certificate would be one step towards trying to step out a little more from the regular retail pharmacists, like me, that didn't have residencies. So that's one thing that would be beneficial for an independent [pharmacist] like me who wants to have a niche or do more collaborating.” (Participant 2).*

All participants agreed that they would recommend the primary care certificate program to pharmacists, specifically those aiming to transition into primary care from another setting or those who did not complete residency training as well as current employees, future employees, and job applicants or candidates. Most participants also stated they would sponsor their current pharmacists' pursuit of this certificate, especially as the pharmacist's role continues to evolve.

*“I simply would [sponsor pharmacists' pursuit of this certificate] because I think it's a return on investment. It does bring value to our stores, to our patients, and hopefully lift the profitability of our stores.” (Participant 10).*

### 3.2 Theme 2: pharmacist employers value primary care certificate training experience when making hiring decisions

Employers shared thoughts related to their perceived level of competitiveness in the job market, candidate differentiation, and hiring potential a pharmacist would have due to primary care certificate completion. Participants perceived that pharmacists who completed this training may be viewed as more competitive in the hiring process and the training showed a pharmacist's motivation, dedication, self-directed learning abilities, initiative, innovation, and professional development (e.g., professional character), which impact their job marketability, sets them apart from other applicants, and make them a more desirable candidate for hire. Furthermore, employers mentioned there could be more value brought to the organization or business by employing a pharmacist who completed this primary care certificate, depending on how they intended to apply those skills.

*“I think it's a very, very, very valuable thing. For new graduates who are having a hard time finding jobs, this may be a good route to further education increase their spectrum.” (Participant 11).*

### 3.3 Theme 3: recommended primary care certificate design according to pharmacist employers

Employers stated that the primary care certificate should include training on how to efficiently and effectively triage patients, possessing the knowledge and skills needed to prioritize and solve problems and care for a wide variety of patients and disease states including diabetes, hypertension, hyperlipidemia, anticoagulation, and more. Beyond clinical topics, participants heavily recommended incorporating training in the areas of billing and reimbursement, collaborative practice agreements, and communication. The topic of leadership was favored by a few participants for inclusion as a module topic within the program curriculum; however, the large majority of participants voted against inclusion of this topic compared to other clinical and non-clinical topics. For this reason, leadership was not included as a module topic in the final program curriculum.

Furthermore, participants shared suggestions for the design of the program, including details related to program duration and hours, price, curricular topics, content delivery, and assessment methods to ensure program competencies are met and learners are equipped with the necessary knowledge and skills to practice in outpatient primary care. All employers agreed there should be a combination of both knowledge- and practice-based learning, with emphasis on the importance of a hands-on experiential component to emphasize and apply what was learned through the didactic portion. Participants shared their perceptions of an ideal breakdown between self-paced, online home study modules and live, experiential learning.

*“I think experiential assessments are paramount. I don't think it's exclusively about knowledge. There are plenty of people who can take an exam and not take care of a patient. So, you need to be able to prove at the end of whatever the thing is that you can do what the paper says you can do. So, there would be some OSCE or some evaluation that shows a skill as well as the knowledge.”*

*(Participant 13)*

These results driven from the focus group data reveal that pharmacist employers perceive primary care certificate training as valuable for pharmacists, helping them succeed in shifting roles and responsibilities within the profession and providing more opportunities for pharmacists in a competitive job market. The results also provided employer recommendations for primary care certificate program curriculum and design. The results of this study informed the evidence-based development of the new Pharmacist Primary Care Certificate Training Program (Table 3) which is now offered by the study institution and accredited by the Accreditation Council of Pharmacy Education. Based on employer input, the design of the program includes 30 h of training over 12 continuous weeks, which count for 30 h of CPE: 20 h are dedicated to didactic learning and are offered via 10 web-based home study modules while the remaining 10 h consist of experiential learning gained through practical skills application via four live simulation assessments. The program is completely virtual in consideration of the scheduling constraints and learning needs of busy pharmacists, as employers emphasized.

**Table 3:** Characteristics of the newly developed pharmacist primary care certificate training program.

Accredited by the Accreditation Council for Pharmacy Education (ACPE)
12 weeks
30 h of CPE credit: 20 h of didactic learning via web-based home study modules +10 h of experiential learning and practical skills application via live virtual simulation
Clinical Coach mentor designated per each pharmacist enrollee
10 home study module topics developed by content experts: • Collaborative Practice Agreements • Billing and Reimbursement • Medication Management • Communication and Teamwork • Hypertension • Dyslipidemia • Diabetes • Anticoagulation • Chronic Lung Disease • Acute Outpatient Care
4 live virtual simulations: • Medication Management • Triage and Treat – Disease State Management (2) • Collaborative Practice Agreement Pitch to a Provider Panel
Learners assessed via completion of all online modules, case studies, and simulations (assessed via rubric)
Certificate and badge of completion

## 4 Discussion

Pharmacist employers perceived primary care certificate training as valuable, helping pharmacists sustain shifting roles and responsibilities, increasing opportunities in a competitive job market, and favorably influencing the hiring decision. As the role of the pharmacist continues to evolve, a primary care certificate training program may prepare pharmacists to engage in direct patient care and develop skills and expertise necessary to succeed in outpatient primary care.

Specific to the pharmacy profession, evolving patient care responsibilities have heightened the requisite skills needed by pharmacists to deliver clinical services. Learners must be prepared for systematic outcomes focused on lifelong learning and continuing professional development. Certificate programs are one way to meet this need and are growing in number. For example, the American Society of Health Systems Pharmacists currently offers 30 certificate programs with two more launching in 2022 and 2023.^13^ These certificate programs are of high quality, easily accessible, and span a variety of relevant topics and skills, factors that may appeal to practicing healthcare professionals.

Skills-based online and hybrid educational opportunities are on the rise including certificate programs, micro-credentialing, boot camps, and credentialing programs.^23^ Assessment of current literature conveys that certificate programs are generally beneficial,^15^, ^16^, ^17^ but little research has been conducted to characterize the specific competencies that employers perceive as necessary for practice. The present study details employers' expectations for pharmacists who engage in certificate training programs. Other research has assessed the effectiveness of a teaching certificate program regarding academic pharmacy and preceptor development that was offered to practicing pharmacists and pharmacy residents.^16^ Their findings suggested that a 10-month certificate program utilizing a combination of didactic strategies and live applications is effective in terms of improving pharmacists' knowledge and confidence of the topics discussed.^16^ Similarly, in another recent study regarding an anticoagulation certificate program, 90% of participants agreed that the presented lecture material was relevant and that the program objectives were met.^24^ Not only did this program meet its goals, but participants expressed that they were able to directly apply this knowledge in anticoagulation clinics and inpatient care settings.^24^

The qualitative findings of the present study informed the development of the new Pharmacist Primary Care Certificate Training Program currently offered at the study institution. At the time this study was conducted, there were no other pharmacist primary care certificate training programs available. Pharmacist employers indicated the need for a certificate training program that included didactic and experiential training and provided the skills needed to prioritize and solve problems and care for a wide variety of patients. The Pharmacist Primary Care Certificate Training Program was designed to meet these overarching goals including case-based didactic content and summative simulations covering both clinically focused disease states and practice management topics.

There are some study limitations to consider. First, because recruitment was from a CPD email listserv, there is potential bias that most of these people value training more than those that do not subscribe to the listserv, though it was not asked whether the focus group participants completed additional training themselves. Second, this research explored employer perceptions of a primary care certificate's impact on their hiring decision, not hiring actions or offers made. In terms of improving candidate marketability to employers, many factors are considered when hiring pharmacist candidates, and preferred characteristics of the candidates can vary depending on the practice settings and the responsibilities that the pharmacist will have if they are hired.^25^^,^^26^ Although a primary care certificate could potentially make a candidate more competitive for a job position, the pharmacist hiring process is multi-faceted.^27^^,^^28^ The most influential criteria for hiring staff pharmacists include licensure status, salary requirements, date of availability, interest in the position, ability to relocate, willingness to work unexpected hours, appearance, honesty, friendliness, police record, and reason for leaving the previous job.^29^ Hiring a new pharmacist requires holistic consideration of each candidate, and most pharmacy employers will view the completion of a primary care certificate as an asset in combination with other desirable characteristics of the pharmacist candidate.^30^^,^^31^ Third, it may be perceived that designing such a certificate program would require extensive work and seem difficult to implement. While the authors can attest to the time, rigor, and effort required in conducting this research and subsequently designing and implementing a pharmacist primary care certificate training program based on employer perceptions, it is extremely achievable with a clear timeline, objectives, necessary resources (e.g., learning platform, content experts, accreditation, etc.) and teamwork. Future research should explore the impact of the newly developed primary care certificate on outcomes such as employer hiring decisions, pharmacist job status, clinical service opportunities, collaborative practice, and patient health.

## 5 Conclusions

Pharmacist employers from a variety of specialties expressed during focus groups that there is a significant need for primary care certificate training program based upon the changing roles and responsibilities that pharmacists are experiencing. Employers stated that the design of the program should utilize a combination of didactic training and hands-on learning experience to provide participants with a more realistic ability to incorporate this training into their daily practice yet also consider the scheduling constraints and learning needs of busy pharmacists. The results of this study informed the development of the new Pharmacist Primary Care Certificate Training Program currently offered by the study institution and accredited by the Accreditation Council of Pharmacy Education. These findings may be used in scaling this program and future primary care certificate training programs to prepare pharmacists to engage in direct patient care and develop the skills and expertise necessary to succeed in the outpatient primary care setting.

## Funding

This research did not receive any specific grant from funding agencies in the public, commercial, or not-for-profit sectors.

## CRediT authorship contribution statement

**Kelsey D. Frederick:** Conceptualization, Methodology, Formal analysis, Investigation, Resources, Data curation, Writing – original draft, Writing – review & editing, Visualization, Project administration. **Rachel E. Barenie:** Conceptualization, Methodology, Writing – original draft, Writing – review & editing, Visualization. **M. Braden Dill:** Formal analysis, Writing – original draft. **James S. Wheeler:** Conceptualization, Methodology, Visualization, Writing – original draft, Writing – review & editing, Supervision.

## Declaration of Competing Interest

None.

## Appendix A Supplementary data

Focus Group Interview Guide Image 1
