# Supplementary material for: Designing a pharmacist primary care certificate training program based on employer perceptions
Source: Explor Res Clin Soc Pharm. 2022 Oct 10;8:100191. doi: 10.1016/j.rcsop.2022.100191 (PMC9579043; doi:10.1016/j.rcsop.2022.100191)
Supplement: Supplementary file 1 — Focus Group Interview Guide [file mmc1.docx]

Designing a pharmacist primary care certificate training program based on employer perceptions

**Appendix A. Employer Focus Group Guide**

1. Describe your organization/practice site and your role in the hiring capacity.
2. In your opinion from a hiring perspective, what is the value of pharmacist certificate program training in today’s job market? Please explain.
3. Do you believe completion of certificate program training helps make pharmacists more competitive?
4. Why or why not?
5. If so, how? What type?
6. Are there any noticeable differences in skill level, orientation/training, clinical competence, etc. compared to pharmacists hired without post-grad training and/or credentialing?
7. The XXX Office of Continuing Professional Development is developing a primary care certificate which aims to equip pharmacists to take on new roles and expand service offerings in the primary care setting. In your opinion, what are the most important primary care skills to include in this certificate program? (competencies expected for graduates to achieve and be practice-ready)
   - 1. Please explain.
8. How would you recommend these skills be taught/developed/assessed ensuring these competencies are achieved? Please explain.
   - 1. How would you weigh these? In other words, what would be the percent breakdown of these activities?
9. How many total earned hours do you believe are necessary for a primary care certificate program to favorably influence your decision to hire a pharmacist? Please explain.
10. What would be a reasonable duration/time-to-completion for a pharmacist primary care certificate training program? Please explain.
11. Would you recommend that future pharmacist applicants pursue primary care certificate training? Why or why not?
12. Would your organization be willing to sponsor/invest in pharmacists’ pursuit of this primary care certificate training? Why or why not?
    - 1. If not, what would the program need to look like for your organization to invest?
13. What other recommendations do you have for a pharmacist primary care certificate program?
14. Is there anything else you’d like to add before we conclude?
